# Impact of the new hazard classes in the CLP regulation on EU chemicals legislation

**DOI:** 10.1186/s12302-025-01054-4

**Published:** 2025-01-29

**Authors:** Diana Kättström, Anna Beronius, Urban Boije af Gennäs, Christina Rudén, Marlene Ågerstrand

**Affiliations:** 1 Department of Environmental Science, Stockholm University, Stockholm, Sweden; 2 Institute of Environmental Medicine, Karolinska Institute, Stockholm, Sweden; 3 Swedish Chemicals Agency, Sundbyberg, Sweden

**Keywords:** CLP, New hazard classes, PBT/vPvB, EDC, PMT/vPvM, Endocrine disruptor

## Abstract

The EU recently introduced four new hazard classes to the Classification, Labelling and Packaging Regulation (CLP) (EC) 1272/2008. The classes are endocrine disruption for human health (ED HH) and the environment (ED ENV), persistent, bioaccumulative and toxic (PBT) or very persistent and very bioaccumulative (vPvB), and persistent, mobile and toxic (PMT) or very persistent and very mobile (vPvM). This action was a direct consequence of the EU’s Chemicals Strategy for Sustainability, which aims at strengthening the protection of human health and the environment, as well as reinforcing the CLP Regulation as the central piece of the chemicals legislation. This study examined the regulatory obligations triggered by these new hazard classes, as well as the existing obligations for endocrine disrupters and PBT/vPvB substances identified in other EU regulations. In addition, we compared the CLP criteria for endocrine disruption and PBT/vPvB to criteria existing in other EU regulations and investigated how these criteria are used in the EU chemicals legislation. We found that the implementation of the new hazard classes under the CLP into existing EU chemicals legislation will require the revision of all regulations that rely on the CLP hazard criteria for risk management. Without revision, the immediate impact of the new hazard classes will only extend to six regulations and the regulatory obligations they contain, all of which apply to substances classified under any of the CLP hazard classes. Meanwhile, substances with endocrine disrupting and PBT/vPvB properties are already being identified and regulated using criteria from regulations other than the CLP. When comparing the criteria for identification of endocrine disrupters and PBT/vPvB substances across the chemicals legislation, we found that the criteria differed between regulations. The findings aim to support the efficient implementation of the new CLP hazard classes and harmonization of criteria across regulations, in line with the Chemicals Strategy for Sustainability.

**Supplementary Information:**

The online version contains supplementary material available at 10.1186/s12302-025-01054-4.

## Introduction

To ensure safe use and to facilitate international trade with chemicals, the United Nations developed the Globally Harmonized System of Classification and Labelling of Chemicals (GHS) [1]. The GHS harmonizes the way hazards of chemicals and mixtures are identified and communicated by providing hazard criteria for the classification of chemicals and mixtures, and hazard communication elements, such as pictograms, signal words and hazard statements. The EU has adopted the GHS and implemented it through Regulation (EC) 1272/2008 on the classification, labelling, and packaging of substances and mixtures (CLP).

Until recently, the CLP included the same hazard classes as the GHS. However, in line with the European Commission's goal to enhance the protection of human health and the environment, four new hazard classes were introduced to the CLP. This amendment marks a step towards establishing the CLP Regulation as a central piece of EU chemicals legislation, as outlined in the Chemicals Strategy for Sustainability [2]. The new hazard classes are: endocrine disruption for human health (ED HH) and the environment (ED ENV), persistent, bioaccumulative and toxic, very persistent and very bioaccumulative (PBT/vPvB), persistent, mobile and toxic, and very persistent and very mobile (PMT/vPvM) [3, 4]. The obligation to apply the new hazard criteria when classifying new substances and mixtures will be mandatory for manufacturers and importers on May 1st, 2025, and for downstream users on May 1st, 2026 [3]. For existing substances and mixtures, the implementation is extended by a transition period of 18–24 months [3].

The main reason for introducing new hazard classes to the CLP, thus diverging from the GHS, was the lack of coherence in the identification, assessment and management of substances with endocrine disruptive and PBT/vPvB properties across EU chemicals legislation [5, 6]. Namely, the criteria for endocrine disruption and PBT/vPvB properties, as well as risk management measures, were defined in other pieces of legislation (further referred to as non-CLP criteria). For example, Annex XIII to REACH provides criteria for the identification of the PBT/vPvB properties, whereas criteria for endocrine disruption are found in the Biocidal Products Regulation and Plant Protection Products Regulation [7–9]. However, without classification criteria in the CLP, the hazards associated with these substances are not efficiently communicated to downstream users and consumers [6]. Furthermore, substances with endocrine-disruptive and PBT/vPvB properties currently do not trigger any regulatory obligations in regulations and directives with established obligations for CLP-classified substances [6]. The criteria for PMT/vPvM properties were not previously included in the EU chemicals legislation. However, some have argued that persistence and ability for long-range transport should be considered as an equivalent level of concern to PBT/vPvB properties [10–14]. The PMT/vPvM criteria have, therefore, been developed to further enhance the protection of the environment, in particular water [14].

The CLP Regulation provides criteria for hazard identification and communication. Harmonized classification is a legally binding classification agreed upon at the EU level, while self-classification is performed by manufacturers, importers, or downstream users. The CLP has a downstream impact, including on other legislation that uses the CLP hazard classification as a basis for further risk assessment or risk management measures, such as restriction or prohibition. Understanding how the CLP Regulation interacts with other legislation is important when implementing new hazard criteria and assessing the impact of these changes. The interlinkages between the CLP and other downstream legislation have been mapped out by the studies supporting the EU Fitness Check [15–17].

In our previous study [18], the interlinkages between the CLP and regulations using CLP hazard classes for risk management were mapped out. The results showed that out of 53 pieces of legislation listed in EU Chemicals Legislation Finder (EUCLEF), 19 had obligations for substances classified under the CLP. This study found regulatory obligations for substances classified into the new CLP hazard classes or with endocrine-disrupting, PBT/vPvB or PMT/vPvM properties in much fewer pieces of legislation. Some of the pieces of chemical legislation with no connection to the CLP, such as Persistent Organic Pollutant Regulation, regulated chemicals on a case-by-case basis, adding the specific substances to lists for which regulatory obligations apply. Others, such as the Urban Waste Water Treatment Directive, were instead referring to the aimed quality of the end product. More recently in 2022, the European Commission published a report that provided technical and scientific support to the revision of the CLP [6]. Mohr et al. [19] have conducted a review focusing on the current regulatory landscape for PMT/vPvM substances. However, to the best of our knowledge, a comprehensive account of regulatory obligations that will apply to the new hazard classes, or those that already apply to the substances with endocrine disruptive and PBT/vPvB properties, has not been performed. The objectives of this study were to.

1. assess how the CLP criteria for endocrine disruption and PBT/vPvB properties differ from those in other regulations, here referred to as the non-CLP criteria, and examine how these criteria are used across the EU chemicals legislation
2. map the regulatory obligations triggered by the new CLP hazard classes for endocrine disruption for human health (ED HH), endocrine disruption for the environment (ED ENV), persistent, bioaccumulative and toxic, very persistent and very bioaccumulative (PBT/vPvB), persistent, mobile and toxic, and very persistent and very mobile (PMT/vPvM)
3. identify existing regulatory obligations for substances with endocrine-disrupting properties or PBT/vPvB properties identified based on criteria in other legislation, here referred to as the non-CLP criteria

Taken together, this study contributes to the aims of the Chemicals Strategy for Sustainability by supporting an efficient and full implementation of the new hazard classes and harmonization of the criteria across legislation.

## Methods

The EU Chemicals Legislation Finder (EUCLEF), available through the European Chemicals Agency, was used to identify relevant EU chemicals regulations and directives. The latest consolidated versions of the identified pieces of legislation were accessed through the website Access to European Union Law (EUR-Lex) run by the Publications Office of the EU. First, criteria related to PBT/vPvB, PMT/vPvM and endocrine-disrupting properties were identified and then compared across the regulations.

To identify regulatory obligations that apply to the substances meeting the criteria of the new CLP hazard classes, the search terms “1272/2008”, “hazardous”, and “classifi” were used. In addition, to identify regulatory obligations that specifically concern substances with PBT/vPvB, PMT/vPvM and endocrine-disrupting properties, the documents were searched using the search terms “PBT”, “vPvB”, “PMT”, “bioaccum”, “toxic”, “persist”, “mobil" and “endocrine”.

Regulations and directives that did not contain any of the search terms were excluded from further analysis. Relevant regulatory obligations were extracted from each piece of legislation and compiled into an Excel database. For each regulatory obligation, the following information was collected: article number and name, CLP hazard class or hazardous properties to which regulatory obligations applied and the type of reference (general/specific), the text of the regulatory obligation, and whether or not additional conditions apply for the obligation to be triggered.

## Results

### The use of non-CLP criteria for endocrine disruption and PBT/vPvB across chemical legislation

**Fig. 1:**
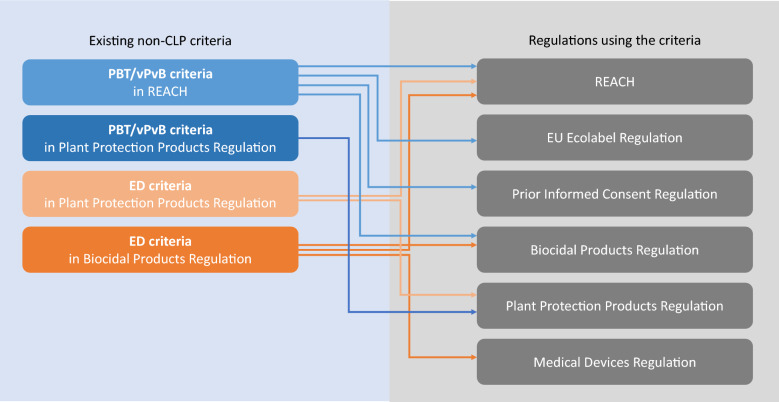
Existing non-CLP criteria for endocrine-disruptive and PBT/vPvB properties and their use by different chemicals legislations.

In addition to the criteria recently introduced in the CLP Regulation, non-CLP criteria for endocrine-disrupting and PBT/vPvB properties have been found in other pieces of chemicals legislation. Non-CLP criteria for PBT/vPvB properties are found in the Plant Protection Products Regulation and REACH, while criteria for endocrine-disruptive properties are found in the Plant Protection Products Regulation and the Biocidal Products Regulation. Figure 1 illustrates the use of the above criteria by other chemicals regulations.

The REACH criteria for PBT/vPvB properties are used by REACH itself, the Biocidal Products Regulation, the EU Ecolabel Regulation, and the Prior Informed Consent Regulation, while the Plant Protection Products Regulation uses its own criteria for the identification of PBT/vPvB properties. The criteria for endocrine disruption, outlined in the Biocidal Products Regulation, is used by the Biocidal Products Regulation itself, the Medical Devices Regulation, and REACH. Similarly, the criteria under the Plant Protection Products Regulation are applied by the regulation itself and by REACH.

The In Vitro Diagnostic Medical Devices Regulation (not shown in Fig. 1) does not refer to any of the criteria; however, the regulation has obligations for substances with endocrine-disrupting properties for which there is scientific evidence of probable serious effects on human health, and which are identified following the procedure set out in Article 59 of REACH. Article 59 of REACH sets out the procedure for the identification of Substances of Very High Concern (SVHC) and the establishment of the candidate list for inclusion on the Authorisation List in Annex XIV. However, substances can only be added to the candidate list if they meet the criteria set out in Article 57 of REACH and an evaluation process has been completed.

### Comparing criteria for ED, PBT/vPvB, and PMT/vPvM

#### Criteria for endocrine-disrupting properties

The criteria used to identify endocrine disruptors for human health and the environment are identical in both the Biocidal Products Regulation and the Plant Protection Products Regulation (Tables 1 and 2). Meanwhile, the criteria for endocrine disruptors of Category 1 under the CLP Regulation are slightly different.

**Table 1:** Criteria for endocrine disruption for human health as stated in Regulation (EC) No 1272/2008 on classification, labelling and packaging of substances and mixtures (CLP), Regulation (EU) No 528/2012 concerning the making available on the market and use of biocidal products (BPR), and Regulation (EC) No 1107/2009 concerning the placing of plant protection products on the market (PPPR)

	CLP	BPR	PPPR
	Annex I, 3.11	As set by the Delegated Regulation (EU) 2017/2100	Annex II, 3.6
Endocrine disruption for human health	3.11 Endocrine disruption for human health CATEGORY 1 Known or presumed endocrine disruptors for human health The classification in Category 1 shall be largely based on evidence from at least one of the following: a) human data; b) animal data; c) non-animal data providing an equivalent predictive capacity as data in points a or b. Such data shall provide evidence that the substance meets all the following criteria: (a) endocrine activity; (b) an adverse effect in an intact organism or its offspring or future generations; (c) a biologically plausible link between the endocrine activity and the adverse effect. However, where there is information that raises serious doubt about the relevance of the adverse effects to humans, classification in Category 2 may be more appropriate CATEGORY 2 Suspected endocrine disruptors for human health A substance shall be classified in Category 2, where all the following criteria are fulfilled: (a) there is evidence of: i. an endocrine activity; and ii. an adverse effect in an intact organism or its offspring or future generations; (b) the evidence referred to in point (a) is not sufficiently convincing to classify the substance in Category 1; (c) there is evidence of a biologically plausible link between the endocrine activity and the adverse effect	Section A—Endocrine-disrupting properties with respect to humans (1) A substance shall be considered as having endocrine-disrupting properties that may cause adverse effect in humans if, based on points (a) to (d) of point (2), it is a substance that meets all of the following criteria, unless there is evidence demonstrating that the adverse effects identified are not relevant to humans: (a) it shows an adverse effect in an intact organism or its progeny, which is a change in the morphology, physiology, growth, development, reproduction or life span of an organism, system or (sub)population that results in an impairment of functional capacity, an impairment of the capacity to compensate for additional stress or an increase in susceptibility to other influences; (b) it has an endocrine mode of action, i.e., it alters the function(s) of the endocrine system; (c) the adverse effect is a consequence of the endocrine mode of action	From 10 November 2018, an active substance, safener or synergist shall be considered as having endocrine disrupting properties that may cause adverse effect in humans if, based on points (1) to (4) of the sixth paragraph, it is a substance that meets all of the following criteria, unless there is evidence demonstrating that the adverse effects identified are not relevant to humans: (1) it shows an adverse effect in an intact organism or its progeny, which is a change in the morphology, physiology, growth, development, reproduction or life span of an organism, system or (sub)population that results in an impairment of functional capacity, an impairment of the capacity to compensate for additional stress or an increase in susceptibility to other influences; (2) it has an endocrine mode of action, i.e., it alters the function(s) of the endocrine system; (3) the adverse effect is a consequence of the endocrine mode of action

**Table 2:** Criteria for endocrine disruption for the environment as stated in Regulation (EC) No 1272/2008 on classification, labelling and packaging of substances and mixtures (CLP), Regulation (EU) No 528/2012 concerning the making available on the market and use of biocidal products (BPR), and Regulation (EC) No 1107/2009 concerning the placing of plant protection products on the market (PPPR)

	CLP	BPR	PPPR
	Annex I, 4.2	As set by the Delegated Regulation (EU) 2017/2100	Annex II, 3.8
Endocrine disruption for the environment	4.2 Endocrine disruption for the environment CATEGORY 1 Known or presumed endocrine disruptors for the environment The classification in Category 1 shall be largely based on evidence from at least one of the following: a) animal data; b) non-animal data providing an equivalent predictive capacity as data in point a. Such data shall provide evidence that the substance meets all the following criteria: (a) endocrine activity; (b) an adverse effect in an intact organism or its offspring or future generations; (c) a biologically plausible link between the endocrine activity and the adverse effect. However, where there is information that raises serious doubt about the relevance of the adverse effects identified at population or subpopulation level, classification in Category 2 may be more appropriate CATEGORY 2 Suspected endocrine disruptors for the environment A substance shall be classified in Category 2, where all the following criteria are met: (a) there is evidence of: i. an endocrine activity; and ii. an adverse effect in an intact organism or its offspring or future generations; (b) the evidence referred to in point (a) is not sufficiently convincing to classify the substance in Category 1; (c) there is evidence of a biologically plausible link between the endocrine activity and the adverse effect	Section B—Endocrine-disrupting properties with respect to non-target organisms (1) A substance shall be considered as having endocrine-disrupting properties that may cause adverse effects on non-target organisms if, based on points (a) to (d) of point (2), it is a substance that meets all of following criteria, unless there is evidence demonstrating that the adverse effects identified are not relevant at the (sub)population level for non-target organisms: (a) it shows an adverse effect in non-target organisms, which is a change in the morphology, physiology, growth, development, reproduction or life span of an organism, system or (sub)population that results in an impairment of functional capacity, an impairment of the capacity to compensate for additional stress or an increase in susceptibility to other influences; (b) it has an endocrine mode of action, i.e., it alters the function(s) of the endocrine system; (c) the adverse effect is a consequence of the endocrine mode of action	From 10 November 2018, an active substance, safener or synergist shall be considered as having endocrine disrupting properties that may cause adverse effects on non-target organisms if, based on points (1) to (4) of the third paragraph, it is a substance that meets all of the following criteria, unless there is evidence demonstrating that the adverse effects identified are not relevant at the (sub)population level for non-target organisms: (1) it shows an adverse effect in non-target organisms, which is a change in the morphology, physiology, growth, development, reproduction or life span of an organism, system or (sub)population that results in an impairment of functional capacity, an impairment of the capacity to compensate for additional stress or an increase in susceptibility to other influences; (2) it has an endocrine mode of action, i.e., it alters the function(s) of the endocrine system; (3) the adverse effect is a consequence of the endocrine mode of action

Under the CLP Regulation, the first criterion requires evidence of “*endocrine activity*”. The equivalent criterion under the Biocidal Products Regulation and the Plant Protection Products Regulation requires that the substance “*has endocrine mode of action, i.e., alters the function(s) of the endocrine system*”. However, the guidance document on the identification of endocrine disruptors under the Biocidal Products Regulation and the Plant Protection Products Regulation states that “*endocrine mode of action*” should be interpreted as “*endocrine activity*” [20].

The second criterion relates to adverse effects. The CLP criterion for both human health and the environment is met if there is evidence of “*an adverse effect in an intact organism or its offspring or future generations*”. Similarly, the criterion for endocrine disruptors for human health in the Biocidal Products Regulation and the Plant Protection Products Regulation applies to “*an intact organism or its progeny*”. However, the Biocidal Products Regulation and the Plant Protection Products Regulation do not explicitly include offspring of the “non-target organisms” in the criteria for endocrine disruptors for the environment.

The third criterion relates to the link between the first two criteria. The CLP criterion requires “*a biologically plausible link between the endocrine activity and the adverse effect*”. In contrast, the Biocidal Products Regulation and the Plant Protection Products Regulation require that “*the adverse effect is a consequence of the endocrine mode of action*”. Similarly to the first criterion, the “*endocrine mode of action*” should be interpreted as “*endocrine activity*” [20].

In addition to the above criteria, the CLP offers a possibility to classify the substance as a known or presumed endocrine disruptor (Category 1), or a suspected endocrine disruptor (Category 2) for human health or the environment. Category 2 classification is recommended if there is evidence of endocrine activity, adverse effect and biologically plausible link but the evidence is not sufficient for classification in Category 1 [4]. The hazard classes for endocrine disruptors under the CLP thus indicate the level of available evidence. The Biocidal Products Regulation and the Plant Protection Products Regulation only provides the possibility to identify if a substance is an endocrine disruptor or not, without different categories indicating level of available evidence or uncertainty.

#### Criteria for PBT/vPvB properties

Apart from the CLP, the criteria for identification of PBT/vPvB properties can be found in REACH and the Plant Protection Products Regulation (Table 3). The vPvB criteria are identical in all three regulations. For the PBT properties, the definitions of persistency (P) and bioaccumulation (B) are also identical. However, the CLP considers the toxicity (T) fulfilled if the substance is an endocrine disruptor for human health or the environment according to hazard classes ED HH or ED ENV of the CLP Regulation.

**Table 3:** Criteria for persistent, bioaccumulative, toxic properties (PBT) and very persistent, very bioaccumulative properties (vPvB) as stated in Regulation (EC) No 1272/2008 on classification, labelling and packaging of substances and mixtures (CLP), Regulation (EC) No 1907/2006 concerning the Registration, Evaluation, Authorisation and Restriction of Chemicals (REACH), and Regulation (EC) No 1107/2009 concerning the placing of plant protection products on the market (PPPR)

	CLP	REACH	PPPR
	Annex I	Annex XIII	Annex II
Persistent, Bioaccumulative and Toxic	4.3.2.1. Classification criteria for PBT A substance shall be considered a PBT substance when it fulfils the persistence, bioaccumulation and toxicity criteria set out in Sects. 4.3.2.1.1 to 4.3.2.1.3 and assessed according to Sect. 4.3.2.3 4.3.2.1.1. Persistence A substance shall be considered to fulfil the persistence criterion (P), where any of the following conditions is met: (a) the degradation half-life in marine water is higher than 60 days; (b) the degradation half-life in fresh or estuarine water is higher than 40 days; (c) the degradation half-life in marine sediment is higher than 180 days; (d) the degradation half-life in fresh or estuarine water sediment is higher than 120 days; (e) the degradation half-life in soil is higher than 120 days 4.3.2.1.2. Bioaccumulation A substance shall be considered to fulfil the bioaccumulation criterion (B), where the bioconcentration factor in aquatic species is higher than 2 000 4.3.2.1.3. Toxicity A substance shall be considered to fulfil the toxicity criterion (T) in any of the following situations: (a) the long-term no-observed effect concentration (NOEC) or ECx (e.g., EC10) for marine or freshwater organisms is less than 0,01 mg/l; (b) the substance meets the criteria for classification as carcinogenic (category 1A or 1B), germ cell mutagenic (category 1A or 1B), or toxic for reproduction (category 1A, 1B, or 2) according to Sects. 3.5, 3.6 or 3.7; (c) there is other evidence of chronic toxicity, as identified by the substance meeting the criteria for classification: specific target organ toxicity after repeated exposure (STOT RE category 1 or 2) according to Sect. 3.9; (d) the substance meets the criteria for classification as endocrine disruptor (category 1) for humans or the environment according to Sects. 3.11 or 4.2	1.1. PBT Substances A substance that fulfils the persistence, bioaccumulation and toxicity criteria of Sects. 1.1.1, 1.1.2 and 1.1.3 shall be considered to be a PBT substance 1.1.1. Persistence A substance fulfils the persistence criterion (P) in any of the following situations: (a) the degradation half-life in marine water is higher than 60 days; (b) the degradation half-life in fresh or estuarine water is higher than 40 days; (c) the degradation half-life in marine sediment is higher than 180 days; (d) the degradation half-life in fresh or estuarine water sediment is higher than 120 days; (e) the degradation half-life in soil is higher than 120 days 1.1.2. Bioaccumulation A substance fulfils the bioaccumulation criterion (B) when the bioconcentration factor in aquatic species is higher than 2 000 1.1.3. Toxicity A substance fulfils the toxicity criterion (T) in any of the following situations: (a) the long-term no-observed effect concentration (NOEC) or EC10 for marine or freshwater organisms is less than 0,01 mg/l; (b) the substance meets the criteria for classification as carcinogenic (category 1A or 1B), germ cell mutagenic (category 1A or 1B), or toxic for reproduction (category 1A, 1B, or 2) according to Regulation EC No 1272/2008; (c) there is other evidence of chronic toxicity, as identified by the substance meeting the criteria for classification: specific target organ toxicity after repeated exposure (STOT RE category 1 or 2) according to Regulation EC No 1272/2008	3.7.2 An active substance, safener or synergist shall only be approved if it is not considered to be a persistent, bioaccumulative and toxic (PBT) substance A substance that fulfils all three of the criteria of the points below is a PBT substance 3.7.2.1. Persistence An active substance, safener or synergist fulfils the persistence criterion, where: — the half-life in marine water is higher than 60 days, — the half-life in fresh or estuarine water is higher than 40 days, — the half-life in marine sediment is higher than 180 days, — the half-life in fresh or estuarine water sediment is higher than 120 days, or — the half-life in soil is higher than 120 days Assessment of persistency in the environment shall be based on available half-life data collected under appropriate conditions, which shall be described by the applicant 3.7.2.2. Bioaccumulation An active substance, safener or synergist fulfils the bioaccumulation criterion, where the bioconcentration factor is higher than 2 000 Assessment of bioaccumulation shall be based on measured data on bioconcentration in aquatic species. Data from both freshwater and marine water species can be used 3.7.2.3. Toxicity An active substance, safener or synergist fulfils the toxicity criterion, where: — the long-term no-observed effect concentration for marine or freshwater organisms is less than 0,01 mg/l, — the substance is classified as carcinogenic (category 1A or 1B), mutagenic (category 1A or 1B), or toxic for reproduction (category 1A, 1B or 2) pursuant to Regulation (EC) No 1272/2008, or — there is other evidence of chronic toxicity, as identified by the classifications STOT RE 1 or STOT RE 2 pursuant to Regulation (EC) No 1272/2008
Very Persistent, Very Bioaccumulative	4.3.2.2. Classification criteria for vPvB A substance shall be considered a vPvB substance when it fulfils the persistence and bioaccumulation criteria set out in Sects. 4.3.2.2.1 and 4.3.2.2.2 and assessed according to Sect. 4.3.2.3 4.3.2.2.1. Persistence A substance shall be considered to fulfil the ‘very persistent’ criterion (vP), where any of the following conditions is met: (a) the degradation half-life in marine, fresh or estuarine water is higher than 60 days; (b) the degradation half-life in marine, fresh or estuarine water sediment is higher than 180 days; (c) the degradation half-life in soil is higher than 180 days 4.3.2.2.2. Bioaccumulation A substance shall be considered to fulfil the ‘very bioaccumulative’ criterion (vB), where the bioconcentration factor in aquatic species is higher than 5 000	1.2. vPvB Substances A substance that fulfils the persistence and bioaccumulation criteria of Sects. 1.2.1 and 1.2.2 shall be considered to be a vPvB substance 1.2.1. Persistence A substance fulfils the ‘very persistent’ criterion (vP) in any of the following situations: (a) the degradation half-life in marine, fresh or estuarine water is higher than 60 days; (b) the degradation half-life in marine, fresh or estuarine water sediment is higher than 180 days; (c) the degradation half-life in soil is higher than 180 days 1.2.2. Bioaccumulation A substance fulfils the ‘very bioaccumulative’ criterion (vB) when the bioconcentration factor in aquatic species is higher than 5 000	3.7.3 An active substance, safener or synergist shall only be approved if it is not considered to be a very persistent and very bioaccumulative substance (vPvB) A substance that fulfils both of the criteria of the points below is a vPvB substance 3.7.3.1. Persistence An active substance, safener or synergist fulfils the ‘very persistent’ criterion, where: — the half-life in marine, fresh- or estuarine water is higher than 60 days, — the half-life in marine, fresh- or estuarine water sediment is higher than 180 days, or — the half-life in soil is higher than 180 days 3.7.3.2. Bioaccumulation An active substance, safener or synergist fulfils the ‘very bioaccumulative’ criterion, where the bioconcentration factor is greater than 5 000

#### Criteria for PMT/vPvM properties

The criteria for PMT/vPvM are only found in the CLP (Table 4). However, the definition of persistent (P) and very persistent (vP) in the PMT criteria are identical to the definition of persistent (P) and very persistent (vP) in the PBT and vPvB criteria under REACH, CLP and the PPP Regulation. In addition, the definition of toxic (T) in the PMT criteria is identical to the definition of toxic (T) in the PBT criteria under the CLP.

**Table 4:** Criteria for persistent, mobile and toxic (PMT), and very persistent, very mobile (vPvM) as stated in Regulation (EC) No 1272/2008 on classification, labelling and packaging of substances and mixtures (CLP)

	CLP
	Annex I
Persistent, Mobile and Toxic	4.4.2.1. Classification criteria for PMT A substance shall be considered a PMT substance when it fulfils the persistence, mobility and toxicity criteria set out in Sects. 4.4.2.1.1, 4.4.2.1.2 and 4.4.2.1.3. and assessed according to Sect. 4.4.2.3 4.4.2.1.1. Persistence A substance shall be considered to fulfil the persistence criterion (P) in any of the following situations: (a) the degradation half-life in marine water is higher than 60 days; (b) the degradation half-life in fresh or estuarine water is higher than 40 days; (c) the degradation half-life in marine sediment is higher than 180 days; (d) the degradation half-life in fresh or estuarine water sediment is higher than 120 days; (e) the degradation half-life in soil is higher than 120 days 4.4.2.1.2. Mobility A substance shall be considered to fulfil the mobility criterion (M) when the log Koc is less than 3. For an ionisable substance, the mobility criterion shall be considered fulfilled when the lowest log Koc value for pH between 4 and 9 is less than 3 4.4.2.1.3. Toxicity A substance shall be considered to fulfil the toxicity criterion (T) in any of the following situations: (a) the long-term no-observed effect concentration (NOEC) or ECx (e.g., EC10) for marine or freshwater organisms is less than 0,01 mg/l; (b) the substance meets the criteria for classification as carcinogenic (category 1A or 1B), germ cell mutagenic (category 1A or 1B), or toxic for reproduction (category 1A, 1B, or 2) according to Sects. 3.5, 3.6 or 3.7; (c) there is other evidence of chronic toxicity, as identified by the substance meeting the criteria for classification as specific target organ toxicity after repeated exposure (STOT RE category 1 or 2) according to Sect. 3.9; (d) the substance meets the criteria for classification as endocrine disruptor (category 1) for human health or the environment according to Sects. 3.11 or 4.2
Very persistent, very mobile	4.4.2.2. Classification criteria for vPvM A substance shall be considered a vPvM substance when it fulfils the persistence and mobility criteria set out in Sects. 4.4.2.2.1 and 4.4.2.2.2 and assessed according to Sect. 4.4.2.3 4.4.2.2.1. Persistence A substance shall be considered to fulfil the ‘very persistent’ criterion (vP) in any of the following situations: (a) the degradation half-life in marine, fresh or estuarine water is higher than 60 days; (b) the degradation half-life in marine, fresh or estuarine water sediment is higher than 180 days; (c) the degradation half-life in soil is higher than 180 days 4.4.2.2.2. Mobility A substance shall be considered to fulfil the ‘very mobile’ criterion (vM) when the log Koc is less than 2. For an ionisable substance, the mobility criterion shall be considered fulfilled when the lowest log Koc value for pH between 4 and 9 is less than 2

### Regulatory obligations

In total, EUCLEF listed 59 pieces of chemicals legislation, excluding the CLP Regulation. Six of these were no longer in force. All examined regulations and directives together with the regulatory obligations may be found in the Supplementary Data file. Table 5 lists legislation that contained regulatory obligations for substances or mixtures either meeting the criteria for hazard classes ED HH, ED ENV, PBT/vPvB or PMT/vPvM under the CLP, and/or those with endocrine disruptive, PBT/vPvB and PMT/vPvM properties according to the non-CLP criteria.

**Table 5:** Regulations and directives that contain regulatory obligations for substances or mixtures meeting the criteria for hazard classes ED HH, ED ENV, PBT/vPvB or PMT/vPvM under the CLP or those with endocrine disruptive, PBT/vPvB or PMT/vPvM properties according to the non-CLP criteria

*Legislation name and number* *Document number*	CLP criteria				Non-CLP criteria	
	ED HH	ED ENV	PBT/vPvB	PMT/vPvM	ED	PBT/vPvB
Biocidal Products Regulation, (EU) 528/2012 02012R0528-20220415	Yes*	Yes*	Yes*	Yes*	Yes	Yes
Chemical Agents Directive, 98/24/EC 01998L0024-20190726	Yes*	No	No	No	No	No
EU Ecolabel Regulation, (EC) 66/2010 02010R0066-20171114	No	Yes*	Yes*	Yes*	No	No
Industrial Emissions Directive, 2010/75/EU 02010L0075-20110106	Yes*	Yes*	Yes*	Yes*	No	No
In Vitro Diagnostic Medical Devices Regulation, (EU) 2017/746 02017R0746-20230320	No	No	No	No	yes	No
Medical Devices Regulation, (EU) 2017/745 02017R0745-20230320	No	No	No	No	Yes	No
Prior Informed Consent Regulation, (EU) 649/2012 02012R0649-20220701	No	No	No	No	No	Yes
Plant Protection Products Regulation, (EC) 1107/2009 02009R1107-20221121	Yes*	YES*	YES*	Yes*	Yes	Yes
REACH—Registration, Evaluation, Authorisation and Restriction of Chemicals Regulation, (EC) 1907/2006 02006R1907-20230629	Yes*	Yes*	Yes*	Yes*	Yes	Yes
Water Framework Directive, 2000/60/EC 02000L0060-20141120	No	No	No	No	No	Yes

Document number relates to the latest consolidated version examined. Regulations marked with an asterisk * have obligations that apply to all CLP hazard classes, i.e., they also apply to substances that meet the criteria for the new hazard classes

Overall, the regulations which were examined here either (1) imposed obligations on “hazardous” substances as defined in the CLP Regulation, meaning that these obligations apply to substances classified under any of the CLP hazard classes, such as all human health or environmental classes, (2) imposed obligations on substances meeting the criteria or classified according to the criteria of one or more specific hazard classes, or (3) imposed obligations on substances with hazards identified using the non-CLP criteria found in other regulations.

As the inclusion of the new hazard classes in the CLP Regulation is relatively recent, at the time of writing there are no regulations or directives that contain regulatory obligations specific to the new hazard classes. However, since the Biocidal Products Regulation, the Industrial Emissions Directive, the Plant Protection Products Regulation and REACH have regulatory obligations that apply to substances classified under any of the CLP hazard classes, these regulatory obligations will also apply to substances that meet the criteria for the new hazard classes. Substances meeting the non-CLP criteria for endocrine disruption or PBT/vPvB trigger regulatory obligations in The Biocidal Products Regulation, the Plant Protection Products Regulation and REACH.

In addition, substances meeting the non-CLP criteria for endocrine disruption trigger regulatory obligations in the In Vitro Diagnostic Medical Devices Regulation and the Medical Devices Regulation, whereas substances meeting the non-CLP criteria for PBT/vPvB trigger regulatory obligations in the Prior Informed Consent Regulation and the Water Framework Directive.

#### Substances or mixtures meeting the CLP criteria for ED HH, ED ENV, PBT/vPvB or PMT/vPvM

In total, six pieces of EU chemicals legislation contain regulatory obligations in relation to the CLP. Four of these relates to substances meeting *any of the hazard criteria of the CLP*, thus including the new hazard classes: (1) the Biocidal Products Regulation, (2) the Plant Protection Products Regulation, (3) the Industrial Emissions Directive and (4) REACH.

Furthermore, (5) the Chemical Agents Directive apply to *the physical and human health hazard classes*, and the obligations of (6) the EU Ecolabel Regulation apply to *all environmental hazard classes and some of the human health hazard classes*.

*The biocidal products regulation* does not allow biocidal and plant protection products containing CLP-classified substances to be authorised under a simplified procedure (Article 25).

*The plant protection products regulation* do not allow plant protection products containing CLP-classified substances to be authorised under a simplified procedure (Article 47(1)).

*The industrial emissions directive* mandates that the Member States require regular maintenance and surveillance of measures taken by the industrial site operator to prevent emissions to soil and groundwater and periodic monitoring of soil and groundwater concerning relevant hazardous substances (Article 14). The directive also requires the site operator to submit information about the levels of hazardous substances in soil and groundwater before starting an operation and after the operation ceases (Article 22). In addition, the Industrial Emissions Directive requires the site operator to take actions to ensure the removal, control, containment or reduction of relevant hazardous substances from the site (Article 22).

*REACH* requires a manufacturer or importer of a hazardous substance or mixture to provide a safety data sheet to the recipient of the substance or mixture (Article 31).

*The chemical agents directive* defines “*hazardous chemical agents*” as “*any chemical agent which meets the criteria for classification as hazardous within any physical and/or health hazard classes laid down in Regulation (EC) No 1272/2008 of the European Parliament and of the Council, whether or not that chemical agent is classified under that Regulation*” [21]. The directive requires the employer to assess whether hazardous chemical agents are present in the workplace and, if so, to take preventive measures (Article 4). For example, the employer must establish procedures for dealing with incidents related to the presence of hazardous agents and make information about the procedures available (Article 7). The employer is also obliged to make information about the hazardous agents available to workers (Article 8) and to arrange for appropriate health surveillance of workers if risks related to hazardous agents have been identified (Article 10). The Chemical Agents Directive also places an obligation on the European Commission, mandating that the relationship between the health effects of hazardous chemical agents and occupational exposure should be assessed (Article 3).

*The EU ecolabel regulation* prohibit awarding the Ecolabel to “*goods containing substances or preparations/mixtures meeting the criteria for classification as toxic, hazardous to the environment, carcinogenic, mutagenic or toxic for reproduction (CMR), in accordance with Regulation (EC) No 1272/2008*” (Article 6(6)).

#### Substances or mixtures with ED, PBT/vPvB or PMT/vPvM properties identified using non-CLP criteria

The PMT/vPvM criteria only exist in the CLP, so there were no existing obligations under any of the examined regulations for substances or mixtures with these properties.

Several regulations contain regulatory obligations for substances or mixtures with endocrine-disruptive, and PBT/vPvB properties, identified based on the non-CLP criteria. Of these, the In Vitro Diagnostic Medical Devices Regulation and the Medical Devices Regulation have obligations for endocrine disruptors only, whereas the Prior Informed Consent Regulation and the Water Framework Directive have obligations for PBT/vPvB substances only. The Biocidal Products Regulation, the Plant Protection Products Regulation and REACH have regulatory obligations for both endocrine disruptors and substances or mixtures with PBT/vPvB properties.

In general, substances with endocrine-disrupting or PBT/vPvB properties may not be approved as biocidal active substances under the *Biocidal Products Regulation* (Articles 4 and 5) or active substances in plant protection products under *the plant protection products regulation* (Annex II, Chapter II (3.7.2)). However, under certain conditions, the approval may still be granted. In that case, the approval and renewal period are limited to a maximum of 5 years. For biocidal and plant protection products containing substances with endocrine disrupting or PBT/vPvB properties, Article 23 of the Biocidal Products Regulation and Article 50 of the Plant Protection Products Regulation mandate the competent authorities to conduct a comparative assessment during authorization or renewal. If the assessment shows that safer alternatives exist, that there are no economic or practical disadvantages, and enough products exist to prevent the development of resistance in the target organism, authorities could restrict or prohibit the product's market availability or use. In addition, the Biocidal Products Regulation does not allow authorisation of any biocidal products containing substances with PBT/vPvB or ED properties for use by the general public (Article 19(4)).

*The prior informed consent regulation* prohibits the export of chemicals with PBT/vPvB properties, even if the other conditions for export are met (Article 14(7)).

*REACH* mandates the registrant to perform an extended risk assessment for substances with PBT/vPvB properties during registration (Article 14(4)), and states that such substances should be prioritized for examinations of testing proposals by the European Chemicals Agency (Article 40(1)). Substances with endocrine-disrupting or PBT/vPvB properties may also be candidates for inclusion in the authorisation list in accordance with Article 57 of REACH.

*The medical devices regulation* (Annex I, Chapter II (10.4.1)) only contains obligations for endocrine disruptors with respect to human health and limits the concentrations of these substances in devices to 0.1% weight by weight. In addition, the medical devices regulation also mandates that the instruction for use contains precautions related to materials containing or consisting of endocrine-disrupting substances (Annex I, Chapter III (23.4)).

Similarly, the in vitro* diagnostic medical devices regulation* concerns substances with endocrine-disrupting properties for human health. Annex I, Chapter II (10) of that regulation require the devices to be manufactured in such a way that the risks posed by the endocrine-disrupting chemicals are minimised.

*The water framework directive* requires the Member States to implement measures to progressively reduce pollution from prioritized PBT/vPvB substances, aiming to cease or phase out emissions, discharges, and losses (Article 4(1)). It also requires the European Parliament and the Council, with help from the European Commission, to adopt specific measures focusing on the cessation or phasing out of discharges and emissions of prioritized PBT/vPvB substances (Article 16).

## Discussion

This paper aims to clarify the regulatory obligations triggered by the newly added CLP hazard classes, as well as the regulatory obligations already existing for substances with endocrine-disrupting and PBT/vPvB properties, based on criteria in other regulations. In addition, we compared the CLP criteria for endocrine disruption and PBT/vPvB to those present in other regulations and examined their application across the EU chemical legislation. We found that a complete implementation of the new hazard classes under the CLP will require the revision of all pieces of legislation that rely on the CLP hazard criteria for hazard identification and risk management. In the absence of such a revision, the immediate impact of the new hazard classes will be limited to the regulatory obligations that apply to substances classified under any of the CLP hazard classes. Meanwhile, substances with endocrine-disrupting and PBT/vPvB properties are already identified and subjected to risk management measures, using criteria from regulations other than the CLP. Comparing the criteria for endocrine disruptors and PBT/vPvB substances across chemicals legislations, we found that the criteria differed slightly, and different regulations used different criteria.

The introduction of CLP hazard classes for endocrine disruption, PBT/vPvB, and PMT/vPvM properties represents an important step towards enhancing the protection of human health and the environment from chemicals with such properties. Although it will take years for this change to be fully implemented, some regulatory obligations will automatically apply to substances that are classified in accordance with the new hazard classes. For example, some of the regulatory obligations under the Biocidal Products Regulation apply to all CLP-classified substances, regardless of the hazard class. In those cases, the classification of a substance as an endocrine disruptor for human health or the environment, PBT/vPvB, or PMT/vPvM under the CLP will automatically trigger these regulatory obligations without the need to revise the regulation. Other regulatory obligations of the Biocidal Products Regulation list specific hazard classes for which they apply. Thus, the revision of the provisions will be necessary to extend the obligation to the new hazard classes. It is reasonable that some regulatory obligations, such as additional information requirements, apply to substances classified in any hazard class and that specific targeted obligations apply to particular hazards, such as carcinogenicity or reproductive toxicity. However, this means that the contribution of the new hazard classes to risk reduction will remain limited until full implementation is accomplished.

While the implementation of the new hazard classes is in progress, substances with endocrine-disrupting and PBT/vPvB properties are already regulated in some pieces of legislation, such as the Biocidal Products Regulation and the Plant Protection Products Regulation. However, the existing regulation is based on non-CLP criteria. The comparison of the non-CLP criteria with the CLP criteria revealed minor linguistic differences. For example, for endocrine-disrupting properties, the language of the CLP criteria has been refined to reflect the interpretation of the “endocrine mode of action” adopted under the Biocidal Products Regulation and the Plant Protection Products Regulation. These linguistic differences may also be a reflection of the range of times over which the regulations entered into force.

Regulations with obligations for substances identified as PBT/vPvB or ED based on non-CLP criteria could be revised to extend these obligations to substances classified as PBT/vPvB or ED under the CLP. A related question is whether substances classified as PMT/vPvM under the CLP should also be considered of equal regulatory concern and included in such revisions. For example, the Water Framework Directive (WFD), which sets out rules for the protection of rivers, lakes and groundwater, currently addresses substances of significant concern for water quality. Extending its scope to include PMT/vPvM substances could strengthen its ability to reduce persistent and mobile pollutants that pose risks to water resources. Similarly, the Prior Informed Consent (PIC) Regulation, which regulates the trade and use of hazardous chemicals, could be adapted to ensure that international controls cover not only PBT/vPvB and ED substances, but also PMT/vPvM substances, reflecting their environmental and health risks. These revisions would bring the regulatory framework into line with the evolving scientific understanding of chemical hazards. Our results also revealed two aspects that differ in the criteria themselves. First, the CLP criteria offer the possibility of classifying substances as endocrine disruptors of Category 2, if the evidence is not sufficient for the Category 1 classification, allowing consideration of level of evidence and uncertainty. Second, the PBT criteria in the CLP extended the toxicity criterion (T) to substances with endocrine-disrupting properties, which could potentially increase the number of PBT-classified substances as more substances become classified as endocrine disruptors. While guidance on the application of the new criteria is still being developed and is expected by the end of 2024, it is not clear whether the more comprehensive criteria in the CLP will allow more substances to be classified. Nonetheless, if the revision of regulations using the CLP criteria for risk management measures will introduce obligations for endocrine disruptors of Category 2, the protection of human health and the environment from these substances will most likely be improved.

Endocrine disruption and PBT/vPvB properties have been recognised and addressed for some time in various pieces of legislation, such as REACH and the Biocidal Products Regulation. The CLP hazard criteria for PMT/vPvM substances are, however, newly developed. Since these criteria are new and did not exist in any other legislation prior to the inclusion into the CLP Regulation, there are no pre-existing regulatory obligations specifically targeting those properties. This means that, compared to the endocrine disruptors and PBT/vPvB properties, for which regulatory obligations are already in place while awaiting full implementation of the CLP Regulation, there is currently no general obligation for PMT/vPvM substances, although there are some examples, where mobility is considered. The Biocidal Products Regulation and the Plant Protection Products Regulation consider the potential for long-range environmental transport when approving products. In addition, substances considered to be persistent organic pollutants (POPs) cannot be approved under the Plant Protection Products Regulation. However, a substance is considered a POP if it is persistent, bioaccumulative and has the potential for long-range transport (8). This is in line with a recent review [19] which concluded that the regulation of PMT/vPvM substances is at an early stage and that a strong political commitment is needed to ensure the protection of human health and the environment from these substances.

A report from the German Environmental Agency [22] estimated that 1.9% of the REACH-registered substances (259 of 13,405) met the PMT/vPvM criteria. However, this number may be an underestimation, due to the significant data gaps present in the REACH database, making it impossible to conclude on 67% of the substances (9047 of 13,405). This is concerning, especially since substances with these properties have the potential to have long-lasting and far-reaching impacts on the environment.

With the introduction of the new hazard classes comes the need for generating relevant data that would allow classification. Since the CLP Regulation does not require the generation of new data, the data needs to be required through other regulations, such as REACH. In connection with the revision and amendment of the CLP Regulation, the Commission announced the revision of REACH, originally planned to be finalized by the end of 2023 [23]. However, the revision is delayed with the latest predicted delivery date being in 2025 [24]. Whether current data requirements under REACH is sufficient to classify endocrine disruptors, PBT/vPvB and PMT/vPvM substances remains unclear. Currently, the data requirements increase with the increased annual tonnage of imported or produced substance. It will be crucial to ensure that the data needed for the classification of endocrine disruptors, PBT/vPvB, and PMT/vPvM substances is generated for all tonnage bands when the REACH data requirements are updated.

With the introduction of new CLP hazard classes, it is imperative to approach the revision of criteria, data requirements and regulatory obligations in a synchronised and systematic manner across regulatory frameworks. This approach will promote consistency as well as efficiency of implementation across all relevant legislation. By synchronising these revisions, regulators can streamline processes and minimise potential discrepancies that may arise from inconsistencies between different sets of criteria and requirements. A systematic approach will allow for all aspects to be carefully considered and aligned with the overarching objectives of the CLP Regulation. Ultimately, such synchronised efforts contribute to a more efficient and effective implementation of the new hazard classes, promoting higher safety standards and regulatory compliance.

Apart from regulatory obligations in regulations with risk reduction measures based on the hazard classifications, the CLP also prescribes labelling with information elements, such as pictograms, signal words, and hazard statements, as a way to communicate hazards to the users of chemicals and chemical products. Pictograms, in particular, are designed for immediate recognition by the user to communicate a specific type of hazard. However, the new hazard classes have so far not been assigned any pictograms [4]. The reason for the lack of associated pictograms for the new CLP hazard classes is that these hazard classes are only introduced into the CLP, and not into the GHS. The pictograms are GHS-specific elements, and until the new CLP hazard classes are introduced into the GHS, they will remain without those elements. This means that the users of chemicals or chemical products with endocrine-disrupting, PBT/vPvB and PMT/vPvM properties will lack standardized visual cues to help them understand the associated hazards and risks, and must rely on hazard statements and other labelling elements.

Meanwhile, the effectiveness of the pictograms in communicating hazards is debatable. A survey among EU citizens conducted on request by the European Commission showed that the comprehension of the pictograms varied depending on the pictogram [25]. The “flame” pictogram was understood by 96% of responders, whereas the “exclamation mark” by only 17%. Similarly, Rosette and Ribeiro [26] found that laboratory technicians in higher education institutions were not able to satisfactorily explain the meaning of 4 of 9 pictograms, although the pictograms were the most commonly noticed element on the chemicals label.

With the expansion of the CLP hazard classes and the goal of the Chemical Strategy for Sustainability to make the CLP the centrepiece of the chemicals legislation, additional hazard classes may need to be introduced. Immunotoxicity and neurotoxicity are two hazards that are currently addressed through the hazard classes “specific target organ toxicity” and “reproductive toxicity” under the CLP [2]. However, the development of new hazard classes for these properties should be assessed according to the Chemicals Strategy for Sustainability [2].

The introduction of the new hazard classes into the CLP has initiated discussion on the potential inclusion of them into the GHS [27]. Such a step would contribute to enhanced safety for workers, consumers, and the environment. First, it would ensure consistency in hazard communication globally, making it easier for users to understand and interpret hazard information regardless of their location. Second, ensuring alignment between the CLP hazard classes and the GHS would facilitate trade by avoiding confusion and discrepancies in hazard communication requirements between different regions. In addition, for businesses that operate in multiple jurisdictions, having consistent hazard classification and labelling requirements under both the CLP and the GHS would streamline compliance efforts. It reduces the need for companies to create multiple sets of labels and safety data sheets for different markets. Finally, by harmonizing hazard classification and labelling under the GHS, there is a greater likelihood of consistent and effective risk management practices being implemented worldwide. This contributes to enhanced safety for workers, consumers, and the environment.

## Recommendations

- The revision of criteria, data requirements and regulatory obligations in relevant pieces of legislation should be performed in a synchronized and systematic manner to ensure efficient and effective implementation of the new CLP hazard classes.
- The efforts to integrate the new CLP hazard classes into the GHS framework should persist, as this will further advance international harmonization and elevate the comprehensiveness of hazard communication on a global scale.

## Supplementary Information

Supplementary material 1.

## Data Availability

All data generated or analysed during this study are included in this published article and its supplementary information files.
